# Diverticulum, or not Diverticulum, That Is the Question! Discussing About a Case of Left Ventricular Outpouching Associated With Bicuspid Aortic Valve Assessed by Cardiac Magnetic Resonance

**DOI:** 10.15171/jcvtr.2015.16

**Published:** 2015

**Authors:** Raffaella Capasso, Maria Panelo, Andrea Fiorelli, Iacopo Carbone, Nicola Galea

**Affiliations:** ^1^ Department of Internal Clinical and Experimental Medicine, Second University of Naples, Naples, Italy; ^2^ Department of Cardiology, Son Espases University Hospital, Palma de Mallorca, Balearic Islands, Spain; ^3^ Department of Radiological, Oncological and Pathological Sciences, Sapienza University of Rome, Rome, Italy

**Keywords:** Heart Aneurysm, Diverticulum, Magnetic Resonance Imaging, Aortic Valve, Congenital Heart Disease

## Abstract

Congenital left ventricular outpouchings (LVOs) are infrequent myocardial malformations, comprising various overlapping abnormalities, whose characterization is often intricate in clinical practice using traditional non-invasive techniques. We describe a rare case of LVO associated with bicuspid aortic valve incidentally found in an asymptomatic adult patient. The LVO was located at basal level of the chamber, crescent-shaped with its largest diameter in short-axis view and presented a thin hypo-contractile wall without hyperintense areas on late gadolinium enhanced (LGE) images. This description corresponds to an overlap between usual definition of aneurism, fibrous and muscular diverticulum. The LVO was evaluated according with a classification recently proposed by Malakan Rad. In this case ventricular geometry was not respected, wall thickness was reduced and wall motion compromised therefore corresponding to a small IIc-type, which is considered having the poorest prognosis. Furthermore, the association with bicuspid aortic valve is very unusual. The patient refused surgery and preferred follow-up.

## Introduction

Congenital left ventricular outpouchings (LVOs) are rare primary myocardial malformations, which comprise some overlapping and poorly defined abnormalities. Current definitions of LVOs are not unequivocal and sometimes contradictory since diagnostic criteria are frequently non exhaustive, mixed, not mutually exclusive.^1^

Moreover the lack of a consistent and shared classification of LVOs subtypes prevented from reaching a general consensus on management and treatment strategy.

Recently, a systematic review of 839 cases disclosed the drawbacks of the current definitions and introduced a simple and robust classification for all LVOs.

We describe a case of a LVO associated with bicuspid aortic valve in an asymptomatic adult patient and we evaluated it according to this novel classification proposed by Malakan Rad et al.^1^

## Case Report

A 47-year-old man with septal hypertrophy suspected by echocardiography (ECHO), during a medical checkup before bony marrow donation, was referred to our Department for performing cardiac magnetic resonance (CMR). The patient was asymptomatic and had no significant previous medical history.

Physical examination, electrocardiogram, laboratory tests, chest radiography were unremarkable.

**Figure 1:**
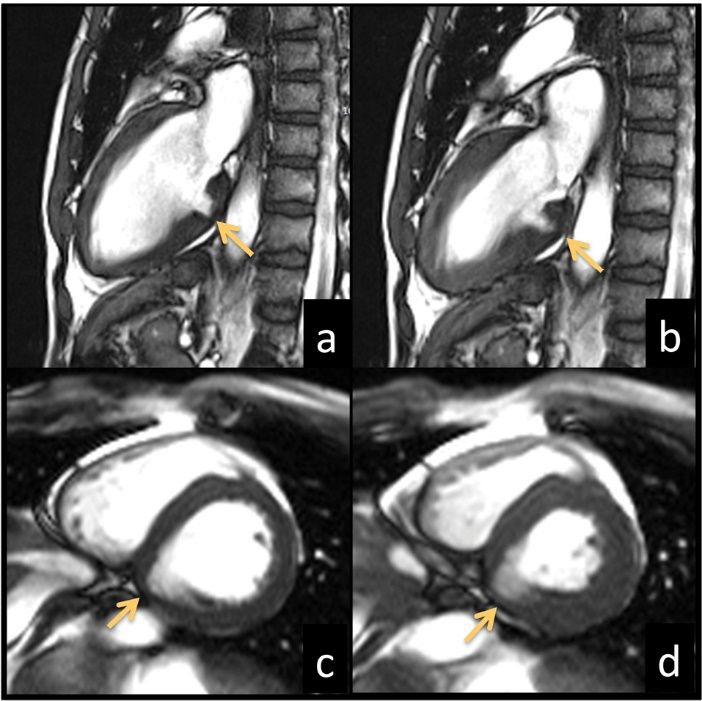
Left ventricle long-axis (a,b) and short-axis (c,d) bright-blood cineMR images acquired in end-diastolic (a,c) and end-systolic (b,d) phases show the presence of an outpouching (arrows) of the inferior ventricular wall at the basal level which does not respect the geometry of the chamber distorting its profile. The abnormality has a crescent-like shape with its largest diameter in the short-axis view. The outpouching wall appears severely hypokinetic with the following dimensions in dyastolic phase: long neck diameter 21 mm, short neck diameter 11 mm, depth 12 mm.

Cine-MRI (Avanto 1.5 T scanner, Siemens, Erlangen, Germany) ruled out myocardial hypertrophy and incidentally revealed an outpouching of the left ventricular inferior wall at the basal level surrounded by a thin layer (4 mm) of hypo-contractile myocardium (Figure 1).

**Figure 2:**
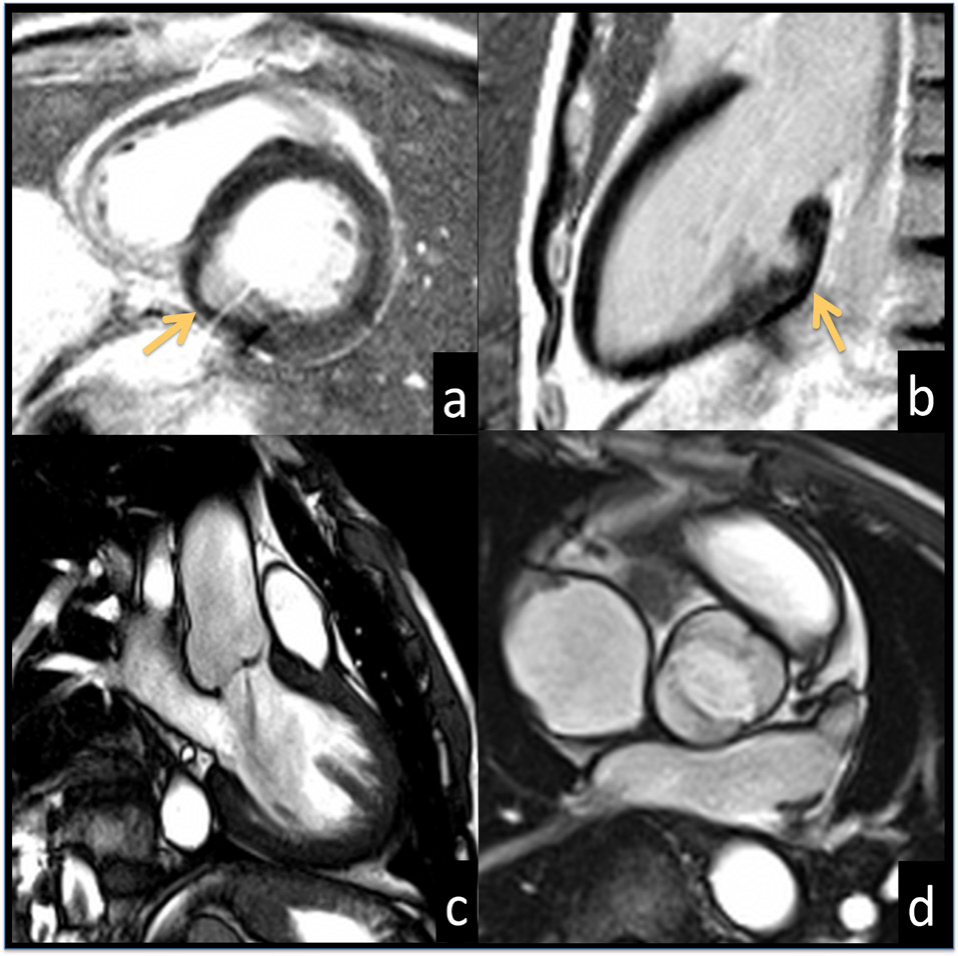
Late gadolinium enhanced images acquired in short-axis (a) and long-axis (b) views reveal no hyperintense signal areas suggesting the absence of fibrous tissue in the ventricular wall (arrow); longitudinal (c) and transverse (d) aortic valve views show a mild jet of regurgitation (c) and the anterior leaflets fusion (d) of the bicuspid valve.

Late gadolinium enhanced (LGE) images showed no hyperintense areas within the LVO myocardial wall (Figure 2a-b).

In addition, a mild regurgitating bicuspid aortic valve was depicted (Figure 2c-d). The patient rejected surgical intervention and opted for follow-up.

## Discussion

Among congenital LVOs, left ventricular diverticulum (LVD) and left ventricular aneurism (LVA) are the most frequent.^1^ LVD is a finger- or hook-like protrusion with narrow connecting neck, a wall composed of all three layers (endocardium, myocardium, epicardium), and systolic contraction synchronous with the ventricular wall.^1-6^ LVD are classified into fibrous and muscular types. The former is prone to rupture and is usually located either in the basal segments or in the subvalvular area. It has a fibrous wall being considered by some authors as a pseudo-diverticulum and not a true diverticulum.^7-9^ Muscular LVD is typically located in the apex, but may also involve the inferior or anterior ventricular walls and it is usually associated with other congenital malformations, particularly with Cantrell’s syndrome.^7,8^

LVA has a broad connecting neck contained by a fibrous ventricular wall, which may show akinesia, dyskinesia, asynchronous contraction, or paradoxical expansion during systole.^1-6^

Although ECHO is the initial diagnostic tool, CMR may play a unique role in differentiating fibrotic from non-fibrotic outpouchings through LGE imaging, and enables a combined evaluation of morphological features, tissue characterization and regional motion.^4,5,7, 10-12^ Preserved contractility defines a LVD while akinetic or dyskinetic motility refers to the presence of fibrosis and may denote either a LVA or a LVD with fibrotic evolution.^11^ The differentiating features of fibrous diverticulum from aneurysm have not been explained in current literature, predominantly based on isolated case reports.^1^

In our patient, LVO was located in basal segment, it had a crescent-like shape and presented an oval neck which appeared larger in short-axis views and narrow in long axis views; residual wall did not show LGE and was markedly hypokinetic.

In clinical practice it is difficult to distinguish non-invasively the different forms of LVOs, because of overlapping imaging features. In our case, LVO showed features of both LVD and LVA, and so it did not correspond to a definite type of LVO making its therapeutic choice questionable.

Malakan Rad et al.^1^ based LVOs classification on three parameters - elliptical left ventricular geometry, wall thickness, and wall motion of the outpouching - offering a prognostic perspective with therapeutical implications.

This new classification introduces the importance of the elliptical geometry that underlines the mechanics of intraventricular blood flow and optimal vortex formation in left ventricle cavity, ignored by the traditional nomenclature.

When the normal elliptical shape of the left ventricular cavity is preserved the LVO is classified as double-chambered left ventricle, and it may be associated with intracavitary obstruction. However, if the elliptical geometry is distorted, but LVO wall thickness and motion are preserved it is classified as type 1, having the best prognosis among all types. If one or both wall features are abnormal, LVO is classiﬁed as type II. Type II is further subclassiﬁed into types IIa (reduced wall thickness), IIb (altered wall motion), and IIc (compromised wall thickness and motion).

In our case elliptical ventricular geometry is not respected, wall thickness is reduced and wall motion compromised then the LVO corresponds to a small IIc type. Type IIc LVOs are considered to have the poorest prognosis, especially if giant.

In our LVO, the broad neck and its dyskinetic wall oriented to surgical resection according to the three cases of small IIc LVOs reported in literature^13-15^, also in relation to the small size and the adult age at diagnosis which are considered to be markers of a good outcome.

Furthermore, our case was associated to bicuspid aortic valve with mild regurgitation and without aortic stenosis.

According to our knowledge only two cases of ventricular outpouching associated with bicuspid aortic valve have been published.^16,17^ Their coexistence could be just a coincidence or their pathophysiology may be related to stretching mechanisms^16^ or to a common congenital structural abnormality.^17^

In particular, it has been hypothesized that a congenital defect of the left ventricular wall near the atrioventricular groove may cause a diverticulum under chronically high intracavitary pressure due to aortic stenosis.^16^

In both published cases, patients underwent cardiac surgery because of their associated pathologies and during the intervention diverticula were removed; pathologic examination confirmed either were fibrous diverticula.

Our case demonstrated that LVO characterization is not as simple as seems and LVO should be analyzed carefully in order to offer to the patient the best therapeutic option based on current evidence.

The classification model proposed by Malakan Rad et al., based only on morphological and functional criteria, is a laudable attempt but needs to be further discussed and refined.

The multiparametric approach offered by CMR may provide additional diagnostic tools, in particular exploiting the capability of LGE imaging to detect the presence of fibrous tissue.

## Ethical Issues

The authors have obtained permission before using patient data and images.

## Competing Interests

Authors declare no conflict of interests in this study.
